# Construction and Validation of a 9-Gene Signature for Predicting Prognosis in Stage III Clear Cell Renal Cell Carcinoma

**DOI:** 10.3389/fonc.2019.00152

**Published:** 2019-03-19

**Authors:** Junlong Wu, Shengming Jin, Weijie Gu, Fangning Wan, Hailiang Zhang, Guohai Shi, Yuanyuan Qu, Dingwei Ye

**Affiliations:** ^1^Department of Urology, Fudan University Shanghai Cancer Center, Shanghai, China; ^2^Department of Oncology, Shanghai Medical College, Fudan University, Shanghai, China

**Keywords:** stage III, clear cell renal cell carcinoma, prognostic model, TCGA, GEO, multi-gene signature

## Abstract

**Purpose:** Aim of this study was to develop a multi-gene signature to help better predict prognosis for stage III renal cell carcinoma (RCC) patients.

**Methods:** Fourteen pairs of stage III tumor and normal tissues mRNA expression data from GSE53757 and 16 pairs mRNA expression data from TCGA clear cell RCC database were used to analyze differentially expressed genes between tumor and normal tissues. Common different expressed genes in both datasets were used for further modeling. Lasso Cox regression analysis was performed to select and build prognostic multi-gene signature in TCGA stage III kidney cancer patients (*N* = 122). Then, the multi-gene signature was validated in stage III renal cancer cases in Fudan University Shanghai Cancer Center (*N* = 77). C-index and time-dependent ROC were used to test the efficiency of this signature in predicting overall survival.

**Results:** In total, 1,370 common different expressed genes were found between tumor and normal tissues in both datasets. After Lasso Cox modeling, nine mRNAs were finally identified to build a classifier. Using this classifier, we could classify stage III clear cell RCC patients into high-risk group and low-risk group. Prognosis was significantly different between these groups in discovery TCGA cohort, validation FUSCC cohort and entire set (All *P* < 0.001). Multivariate cox regression in entire set (*N* = 199) revealed that risk group classified by 9-gene signature, age of diagnosis, pN stage and ISUP grade were independent prognostic factor of overall survival in stage III kidney cancer patients.

**Conclusion:** We developed a robust multi-gene classifier that can effectively classify stage III RCC patients into groups with low and high risk of poor prognosis. This signature may help select high-risk patients who require more aggressive adjuvant target therapy or immune therapy.

## Introduction

Kidney cancer is one of the most common urological tumors worldwide, and nearly 65,340 new cases and 14,970 deaths were estimated in the United States in 2018 (1). The morbidity of renal cell carcinoma (RCC) is also increasing in China (2). At present, prognostic prediction is mainly based on pathological stages of RCC patients (3).

Currently, the tumor node metastasis (TNM) classification system is recommended for tumor staging in clinical practice (4). However, obvious survival differences exist between the subgroups of stage III RCC patients (T3N0M0, 5-year survival: 20–70%; T1-3N1M0, 5-year survival: 0–20%) (5). Although the latest edition (8th) of the UICC/AJCC TNM staging system has been released, this problem remains unsolved, and thus limits the application of this system in estimating prognosis to direct clinical practice. Therefore, better signatures are required to help predict prognosis for stage III RCC patients.

In addition, systemic treatment for patients with stage III RCC is still in development. Several studies have claimed that stage III patients receiving sorafenib or sunitinib after surgery had better disease-free survival (DFS) but a similar overall survival (OS) compared with placebo (6–8). Pazopanib and nivolumab were reported to be effective in metastatic RCC patients, while a clinical trial of pazopanib vs. placebo for adjuvant therapy in locally advanced RCC patients did not show protection (9–12). Adjuvant and neoadjuvant nivolumab clinical trials are ongoing, but hopefully they will show a good response. Use of ipilimumab may also be promising (13, 14). Therefore, a more accurate prognosis classification system for stage III RCC patients is important to direct better management strategies.

Clear cell renal cell carcinoma (ccRCC) is the most common subtype of RCC (15). A study at our center demonstrated that 88.9% of RCC patients have a clear cell subtype in coastal Chinese areas, consistent with results (87.4%) from the SEER (2004–2012) database (16). Furthermore, it was reported that ccRCC patients have more malignant characteristics and worse prognosis. Further advancements are thus urgently required for ccRCC diagnosis and treatment (15). In our study, we focused on prognosis prediction for stage III ccRCC patients and constructed a nine-gene signature, using data from the Gene Expression Omnibus (GEO) and The Cancer Genome Atlas (TCGA) databases. We validated this signature in a cohort of stage III ccRCC patients who underwent radical nephrectomy at Fudan University Shanghai Cancer Center (FUSCC).

## Materials and Methods

### Patients and Public Datasets

Raw microarray mRNA expression data of 14 paired tumor and normal tissue samples from stage III ccRCC patients were downloaded from GEO (http://www.ncbi.nlm.nih.gov/geo/) with the identifier GSE53757. Expression data for a further 16 paired tumor and normal samples from stage III renal cancer patients in TCGA were obtained from UCSC (University of California, Santa Cruz) Xena (https://xenabrowser.net/datapages/). Gene expression data of paired samples from these two datasets were used to identify differentially expressed genes (DEGs) and perform cross-validation to ensure reliability.

At the discovery stage, 122 stage III ccRCC patients with full clinical and survival information along with gene expression data in tumor tissue were included in this study. Data for the discovery stage were used to build a multi-gene signature to predict prognosis in stage III renal cancer cases.

At the validation stage, we recruited 77 patients who underwent radical nephrectomy at Fudan University Shanghai Cancer Center from January 2007 to July 2013 (see detailed information in Supplementary Table 4). These patients all had stage III ccRCC, and total RNA of their tumor tissue were extracted. Data at the validation stage were used to test the efficiency of the multi-gene classifier established at the discovery stage.

For the entire set, 199 stage III renal cancer patients with clinical and gene expression data were included in this study, and all patients exhibited a clear cell pathological phenotype.

### Processing of Public Datasets

Raw microarray data from GSE53757 was produced by the Affymetrix HG-U133 plus 2.0 platform. Data extraction and normalization was conducted using R Bioconductor with Affy and gcrma packages. All probes were mapped based on their Entrez Gene ID. When multiple probes were mapped to the same EntrezGeneID, the mean value was used to represent its average expression level. Gene expression data from TCGA was derived from RNA-sequencing, and pre-processed level 3 data were used in this project.

### Identification of DEGs

Normalized mRNA expression data of 14 paired tumor and normal tissues from GSE53757 were compared using paired *t*-tests to identify DEGs. We undertook a significance analysis of the microarrays, with a false discovery rate of <0.01, *P*-values of <0.01, and fold-changes higher than 2. Level 3 gene expression data from TCGA were also analyzed using the procedures described above. Then, commonly upregulated and downregulated genes in tumor tissue from the two datasets were defined as DEGs. We used MeV version 4.2 to perform the data analysis.

### RNA Extraction, Reverse Transcription, and qRT-PCR Analysis

In the FUSCC validation set, total RNA was isolated from 77 patients' samples using TRIzol reagent (15596-026, Invitrogen). A PrimeScript RP reagent kit (K1622, Thermo Scientific) was used to synthesize first-strand cDNA from total RNA. Then, SYBR Green real-time PCR was performed on the ABI 7900HT platform (Applied Biosystems, USA). We used *ACTB* mRNA as an internal reference. Primers of mRNAs tested in this study were synthesized by Sangon (Shanghai, China) and sequences are listed in Supplementary Tables 1, 2. Gene expression level was presented as ΔCt using the following formula:

$$\begin{array}{l} \Delta \text{Ct}\left(\text{A gene}\right)=\text{Ct (A gene)}-\text{Ct }\left(\text{ACTB}\right) \end{array}$$

Generally, the higher the ΔCt value, the lower the initial gene expression.

### Calculation of Risk Score and Statistical Analysis

Overall survival (OS) was calculated from the date of surgery to the date of death or last follow-up for each cohort. Sex, ISUP (The International Society of Urological Pathology) grade (low vs. high), tumor laterality, pT stage, pN stage, adjuvant target therapy status, and risk group were deemed as categorical variables. Age at diagnosis and risk score were considered as continuous variables. Gene expression levels from GEO or TCGA databases and ΔCt value of certain genes were continuous variables. However, they were divided into high- or low-expression groups to construct and validate the multi-gene prognostic model using best cutoffs estimated by X-tile 3.6.1 (Yale University, New Haven, CT, USA).

After identifying DEGs between tumor and normal tissues, we used LASSO (least absolute shrinkage and selection operator) Cox regression analysis in the discovery stage to select a panel of genes, and then constructed a multi-gene signature for predicting prognosis in stage III ccRCC patients. LASSO Cox regression analysis was performed using the glmnet R package. Detailed R codes and parameters are presented in the Supplementary Files. Patients were divided into high-risk and low-risk groups by a specific risk score formula, using the median risk score in the discovery stage as the cutoff value. OS was calculated and the Kaplan-Meier method was used to test prognostic differences between high- and low-risk groups in the discovery, validation, and entire sets. Cox regression analysis was conducted to test whether risk group was an independent prognostic factor. Time-dependent receiver-operating characteristic (ROC) analysis was performed to assess the predictive accuracy of the risk score in each set. The C-index was calculated to represent the effect of some prognostic factors. A nomogram and related calibration curves were established based on the entire stage III ccRCC cohort for further clinical application. Statistical analyses were performed using R software. All tests were two-tailed, and a *P*-value of <0.05 was considered statistically significant.

## Results

### Clinical Characteristics of Patients in TCGA and FUSCC Cohorts

In TCGA discovery cohort, the median age of stage III patients was 64.5 years (range, 32–88 years). Male patients accounted for 65.6% of the cohort. Patients with ISUP III to IV grade tumors accounted for 71.3%. Among 122 patients, only nine (7.4%) were confirmed to have regional lymph node metastasis according to pathological results. Median follow-up time was 37.8 months.

**Figure 1:**
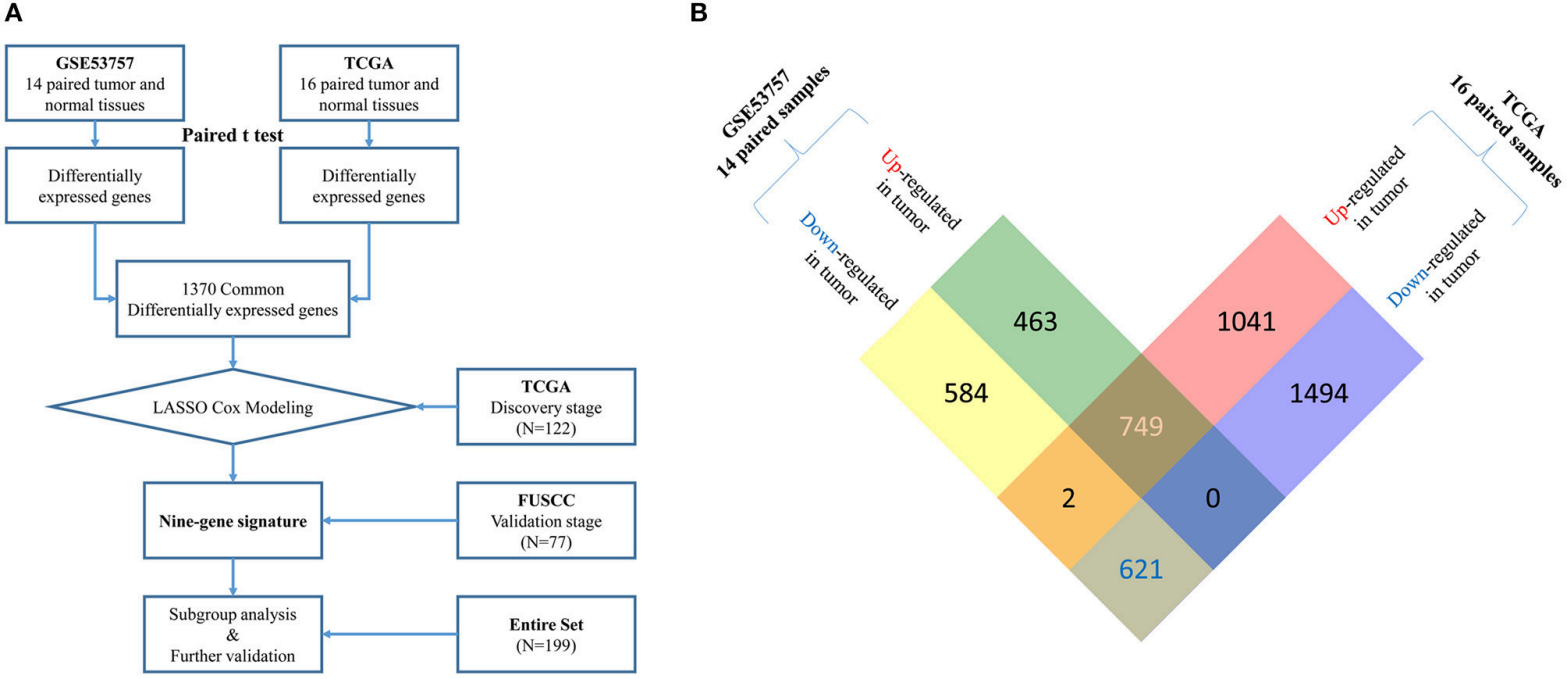
**(A)** Flow chart of multi-gene signature identification and validation.Raw microarray mRNA expression data of 14 and 16 paired tumor and normal tissue samples from stage III ccRCC patients was downloaded from GEO (GSE53757) and TCGA database, respectively. Differentially expressed genes (DEGs) were identified based on gene expression data of paired samples from these two datasets using paired *t*-test. 1370 common DEGs were found between two datasets (see more detail in **B**). Then, we used a LASSO cox regression model and selected nine genes highly associated with survival in these 1370 common DEGs in a TCGA discovery cohort (*N* = 122). Later, this nine-gene signature was further tested in the FUSCC validation cohort (*N* = 77). Finally, subgroup analysis and further validation were performed in the entire cohort (*N* = 199). **(B)** Identification of common up-regulated genes and down-regulated genes (DEGs) from two datasets. By analyzing 14 paired tumor and normal microarray mRNA expression data from stage III ccRCC patients in GSE53757, we identified 1,212 up-regulated and 1,207 down-regulated genes in tumor tissues. From 16 paired samples in TCGA, we identified 1,792 up-regulated and 2,115 down-regulated genes in tumor tissues. After analyzing the intersection of the two datasets, we finally located 749 common up-regulated genes and 621 common down-regulated genes (1,370 common DEGs).

In the FUSCC validation cohort, the median age was 58.0 years (range, 21–82 years). Among 77 stage III patients, 44 were male (57.1%) and 24 patients (31.2%) had lymph node metastasis. Twenty-five patients received adjuvant targeted therapy after surgery. Median follow-up time was 44.5 months. Detailed information of these two cohorts and comparisons between them are summarized in Table 1. A flow chart of our study design is shown in Figure 1A.

**Table 1:** Patient characteristics in discovery stage and validation stage.

**Characteristics**	**TCGA cohort**, ***N*** **=** **122**		**FUSCC cohort**, ***N*** **=** **77**		***P*-value**
	**Median**	**Range**	**Median**	**Range**	
**Age, years**	**64.5**	**32.0–88.0**	**58.0**	**21.0–82.0**	**0.008**
	**Number**	**Percentage, %**	**Number**	**Percentage, %**	
Gender					0.293
Male	80	65.6	44	57.1	
Female	42	34.4	33	42.9	
Living status					0.081
Dead	48	39.3	40	51.9	
Alive	74	60.7	37	48.1	
ISUP grade					0.245
I-II	35	28.7	16	20.8	
III-IV	87	71.3	61	79.2	
T stage					–
T3a	79	64.8	55	71.4	
T3b	37	30.4	6	7.8	
T3c	2	1.6	2	2.6	
T3 unclear	1	0.8	1	1.3	
Others	3	2.4	13	16.9	
N stage					**<0.001**
N1	9	7.4	24	31.2	
N0 or Nx	113	92.6	53	68.8	
Laterality					0.381
Left	53	43.4	39	50.6	
Right	69	56.6	38	49.4	
Adjuvant target therapy					–
Yes	–	–	25	32.5	
No	–	–	52	67.5	
Risk group					0.192
Low risk	61	50.0	31	40.3	
High risk	61	50.0	46	59.7	

*Bold values stand for a p < 0.05*.

### Differentially Expressed Genes (DEGs)

By analyzing 14 paired tumor and normal microarray mRNA expression data from stage III ccRCC patients in GSE53757, we identified 1,212 upregulated and 1,207 downregulated genes in tumor tissues. From 16 paired samples in TCGA, we identified 1,792 upregulated and 2,115 downregulated genes in tumor tissues. By analyzing the intersection of the two datasets, we located 749 commonly upregulated genes and 621 commonly downregulated genes (Figure 1B). These common DEGs were used for construction of the prognostic signature.

### Development of a Multi-Gene Classifier at the Discovery Stage

We used a LASSO Cox regression model to select proper genes highly associated with survival in 1,370 common DEGs in TCGA discovery cohort. LASSO coefficient profiles and a partial likelihood deviance plot are shown in Supplementary Figure 1. Finally, we selected nine genes that were highly associated with prognosis in stage III ccRCC patients (*ATP6V1C2, PCSK1N, PREX1, ANK3, HLA-DRA, SELENBP1, TYRP1, GABRA2*, and *SERPINA5*; see detailed information in Supplementary Table 2). Then we used X-tile to select the optimum cutoff for the expression of these nine genes based on the association with patients' OS in TCGA cohort. After that, the expression level of each gene was divided into high expression (status 1) and low expression (status 0). Then we derived a formula to calculate the risk score for predicting prognosis based on the expression levels of the nine genes in patients (high or low). The formula was as follows: risk score = (0.93^*^SELENBP1 status) + (0.74^*^SERPINA5 status) + (0.39^*^GABRA2 status) + (0.29 TYRP1 status) + (0.02 ATP6V1C2 status) - (1.54 PCSK1N status) – (1.24 PREX1 status) – (0.53 HLA-DRA status) – (0.47 ANK3 status).

Next, patients in the discovery stage were divided into low-risk (*n* = 61) and high-risk (*n* = 61) groups based on the median risk score (−1.73) as a cutoff. To better illustrate this, we adjusted the risk score formula as follows: risk score = (0.93^*^SELENBP1 status) + (0.74^*^SERPINA5 status) + (0.39^*^GABRA2 status) + (0.29 TYRP1 status) + (0.02 ATP6V1C2 status) - (1.54 PCSK1N status) – (1.24 PREX1 status) – (0.53 HLA-DRA status) – (0.47 ANK3 status) + 1.73. Using this formula, a risk score of <0 indicates low-risk while a risk score >1 indicates high-risk.

### Prognostic Value of Nine-Gene Classifier

**Figure 2:**
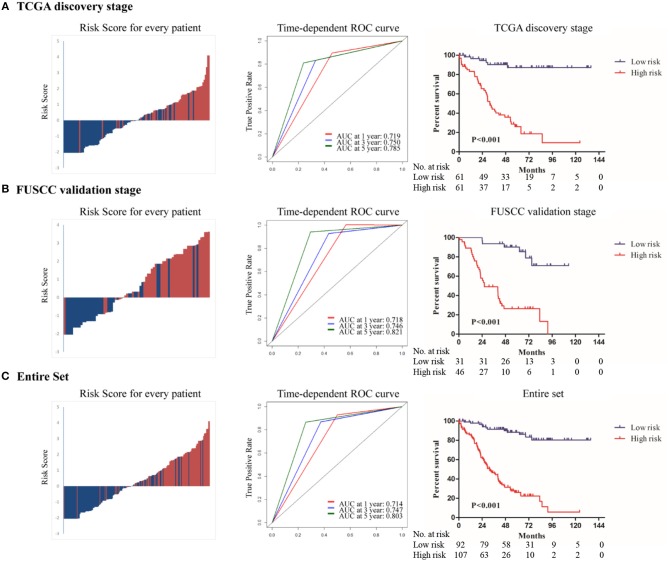
The distribution of risk score, risk group and survival status(left panel); Time dependent ROC analyses at 1, 3, and 5 year after surgery(middle panel); The Kaplan–Meier plot (low risk vs. high risk ccRCC cases) of 5 year overall survival in patients (right panel) in **(A)**TCGA discovery stage, **(B)** FUSCC validation stage, **(C)** the entire set.

The distribution of risk score, risk group, and survival status in the discovery stage is shown in Figure 2A (left panel), which indicated that low-risk patients generally had better overall survival. Time-dependent ROC analyses were performed to evaluate the accuracy of the nine-gene classifier in predicting survival at 1, 3, and 5 years after surgery (Figure 2A, middle panel). A Kaplan-Meier plot indicated that patients in the high-risk group had significantly poor OS with a 5-year survival rate only 22.3% (*P* < 0.001, Figure 2A, right panel).

To confirm whether the prognostic value of the nine-gene signature remained in other datasets, we validated it in the FUSCC stage III ccRCC cohort. Using the pre-established cutoff of risk score in the discovery stage, 31 patients were low-risk and 46 patients were high-risk in the FUSCC validation stage (Table 1). The same analyses were conducted at the validation stage and similar results were achieved. Details are shown in Figure 2B.

In the entire set analysis, the risk score-based classification yielded similar results (Figure 2C). Area under the curve at 1, 3, and 5 years was 0.714, 0.747, and 0.803 in the entire set, respectively. Five-year overall survival rates in low-risk patients and high-risk patients were 86.1 and 26.1%, respectively (*P* < 0.001).

### Independence Analysis and Sub-Group Analysis

To examine whether the nine-gene signature-based risk group classifier was an independent prognostic factor, we performed multivariate Cox regression analysis at the discovery stage and validation stage and in the entire set by adjusting the available clinicopathological variables. We found that risk group (nine-gene signature-based) was an independent prognostic factor at the discovery stage (HR: 10.460; 95% CI: 4.252–25.734; *P* < 0.001), validation stage (HR: 10.204; 95% CI: 3.969–26.234; *P* < 0.001), and entire set (HR: 9.874; 95% CI: 5.234–18.629; *P* < 0.001). Laterality was also an independent prognostic factor (HR: 0.462; 95% CI: 0.251–0.850; *P* = 0.013) in the discovery stage. Additionally, in the entire cohort, we found that age (HR: 1.033; 95% CI: 1.012–1.055), ISUP grade (HR: 2.654; 95% CI: 1.447–4.867), and pN stage (HR: 3.143; 95% CI: 1.891–5.225) remained independent prognostic factors (Table 2).

**Table 2:** Univariate and multivariate Cox regression model in predicting overall survival of stage III clear cell renal cell carcinoma.

**Characteristic**	**TCGA discovery stage**				**FUSCC validation stage**				**Entire set**			
	**Univariate model**		**Multivariate model**		**Univariate model**		**Multivariate model**		**Univariate model**		**Multivariate model**	
	**HR (95% CI)**	***P-*value**	**HR (95% CI)**	***P-*value**	**HR (95% CI)**	***P-*value**	**HR (95% CI)**	***P-*value**	**HR (95% CI)**	***P-*value**	**HR (95% CI)**	***P-*value**
Age	1.043 (1.015–1.072)	**0.003**	1.035 (1.004–1.066)	**0.024**	1.013 (0.982–1.044)	0.432	1.031 (0.998–1.066)	0.068	1.027 (1.007–1.047)	**0.008**	1.033 (1.012–1.055)	**0.002**
**GENDER**												
Male	Reference		Reference		Reference		Reference		Reference		Reference	
Female	0.998 (0.556–1.791)	0.994	0.743 (0.390–1.414)	0.365	1.130 (0.603–2.121)	0.702	1.000 (0.513–1.947)	0.999	1.088 (0.712–1.662)	0.697	0.860 (0.554–1.334)	0.500
**ISUP GRADE**												
I-II	Reference		Reference		Reference		Reference		Reference		Reference	
III-IV	2.093 (0.979–4.471)	0.057	4.251 (1.859–9.718)	**0.001**	3.031 (1.174–7.824)	**0.022**	1.692 (0.624–4.585)	0.301	2.382 (1.321–4.295)	**0.004**	2.654 (1.447–4.867)	**0.002**
**N STAGE**												
N0 or Nx	Reference		Reference		Reference		Reference		Reference		Reference	
N1	2.056 (0.811–5.212)	0.129	3.678 (1.395–9.701)	**0.008**	3.440 (1.830–6.469)	**<0.001**	3.417 (1.756–6.647)	**<0.001**	2.825 (1.749–4.564)	**<0.001**	3.143 (1.891–5.225)	**<0.001**
**LATERALITY**												
Left	Reference		Reference		Reference		Reference		Reference		Reference	
Right	0.530 (0.299–0.939)	**0.030**	0.462 (0.251–0.850)	**0.013**	1.601 (0.853–3.002)	0.143	1.783 (0.902–3.348)	0.098	0.864 (0.569–1.313)	0.495	0.873 (0.565–1.350)	0.542
**ADJUVANT TARGET THERAPY**												
No					Reference		Reference		-		-	
Yes					1.529 (0.800–2.924)	0.199	1.188 (0.599–2.358)	0.622				
**RISK GROUP**												
Low risk	Reference		Reference		Reference		Reference		Reference		Reference	
High risk	10.666 (4.492–25.328)	**<0.001**	10.460 (4.252–25.734)	**<0.001**	7.950 (3.288–19.222)	**<0.001**	10.204 (3.969–26.234)	**<0.001**	9.760 (5.248–18.148)	**<0.001**	9.874 (5.234–18.629)	**<0.001**

*Bold values stand for a p < 0.05*.

**Figure 3:**
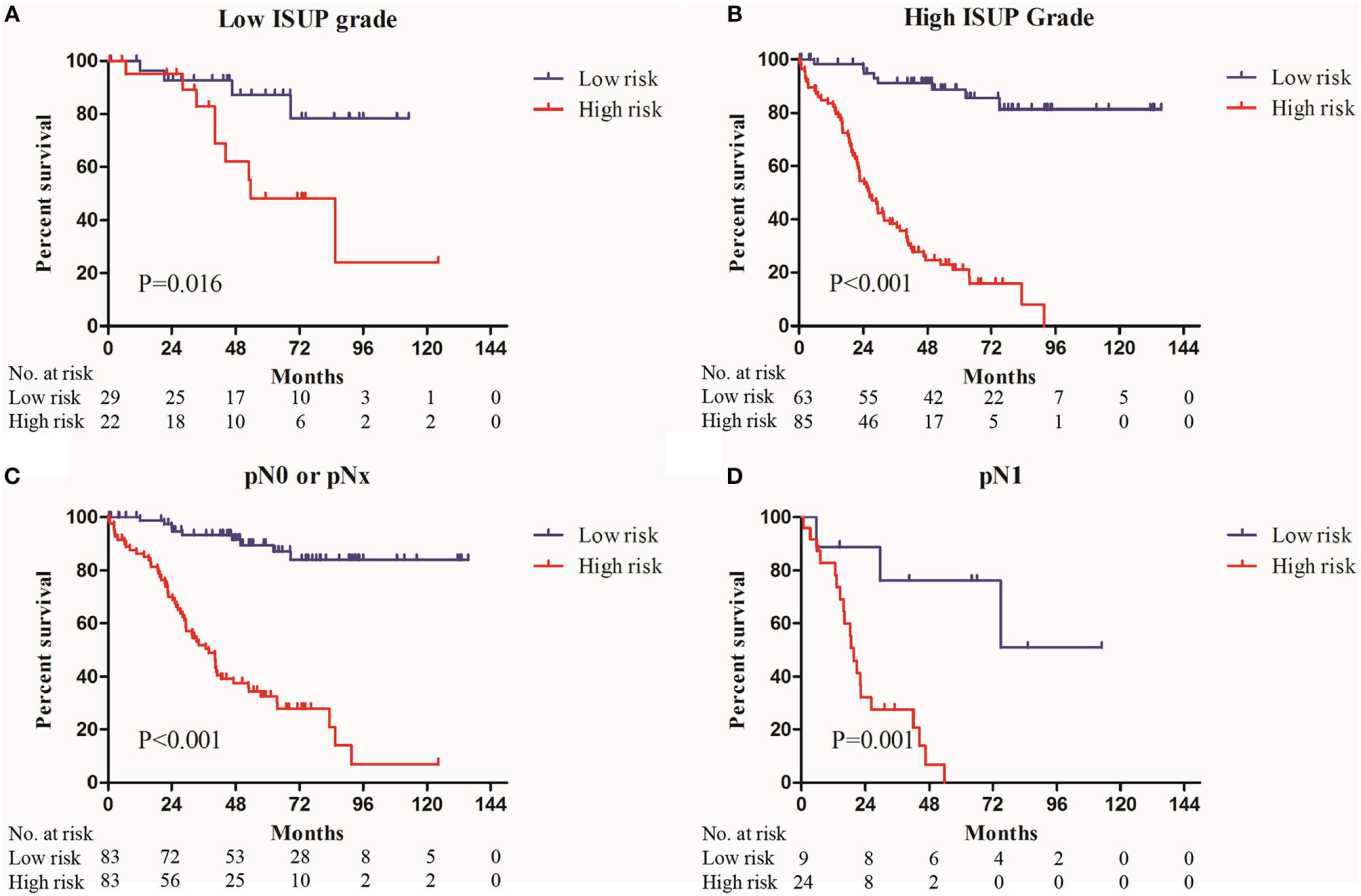
The Kaplan–Meier plot (low risk vs. high risk ccRCC cases) of 5 year overall survival in patients with **(A)** low ISUP grade, **(B)** high ISUP grade, **(C)** pN0 or pNx stage, **(D)** pN1 stage in the entire set.

Sub-group analysis showed that nine-gene signature-based risk group stratification was still a powerful tool in predicting OS in patients with low or high ISUP grade, patients with or without regional lymph node metastasis, older or younger, and male or female patients (Figure 3, Supplementary Figure 2).

### Extension of Prognostic Models for Stage III ccRCC Patients

In the entire cohort, the multivariate Cox regression model revealed that age, ISUP grade, pN stage, and the nine-gene classifier were independent prognostic factors for stage III ccRCC patients. We calculated C-indexes to evaluate the power of these factors. The C-index of the risk group (nine-gene classifier) was 0.719 (95% CI: 0.678–0.761), which was higher than the three clinical factors combined (C-index: 0.690; 95% CI: 0.634–0.746). Then, when we combined the clinical factors and risk group, the C-index increased to 0.792 (95% CI: 0.749–0.835), which showed a better predictive power (Supplementary Table 3).

**Figure 4:**
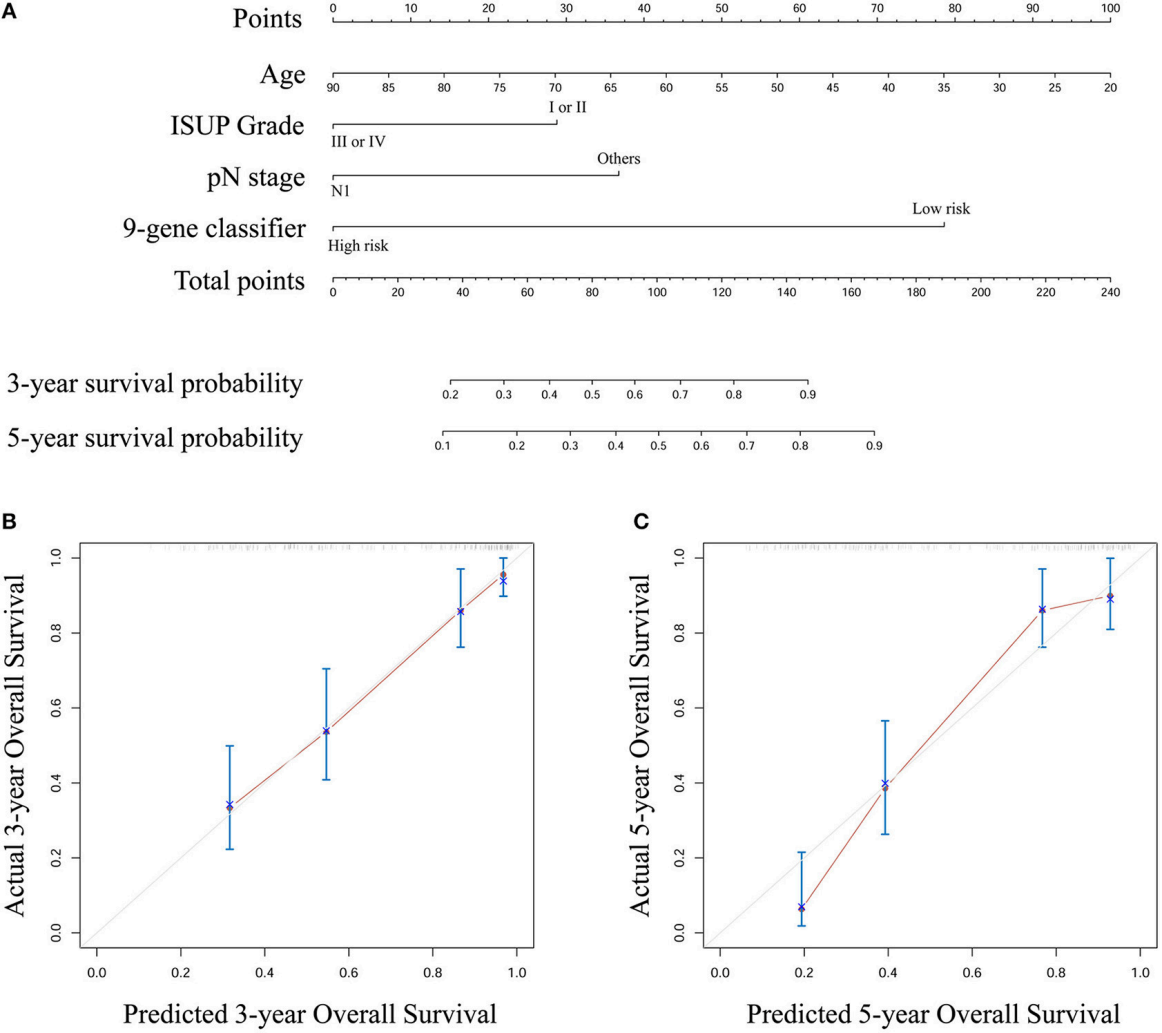
**(A)** A nomogram to predict survival probability at 3 and 5 year after surgery for stage III ccRCC patients based on the results deriving from the entire set **(B)** Calibration curve for the nomogram when predicting 3 year overall survival **(C)** Calibration curve for the nomogram when predicting 5 year overall survival.

Based on the results derived from multivariate Cox regression of OS in the entire set, we developed a nomogram to predict survival probability at 3 and 5 years after surgery for clinical use (Figure 4A). Calibration curves for this nomogram are plotted in Figures 4B,C.

## Discussion

A nine-gene signature was generated using gene expression data from two public databases and was validated in two cohorts of patients with stage III ccRCC. Our results suggested that this model could properly classify patients into different risk groups. Furthermore, this nine-gene signature was also an independent prognosis factor for stage III ccRCC patients, with a better predictive ability than age, ISUP grade, and pN stage. Finally, we developed a nomogram that included these clinical factors and risk group.

Many prognosis models for RCC patients have previously been reported. In 2002, Frank et al. proposed the SSIGN score, which predicts the outcome of patients with ccRCC treated with radical nephrectomy, and is composed of TNM stage (the modified edition in 1997), tumor size, nuclear grade, and necrosis (3). Lam et al. reported a valid prognostic nomogram and risk stratification system in 2005, which was aimed at postoperative surveillance for patients with localized and locally-advanced RCC and included physical examination, complete blood count, serum chemistry, liver function tests, and chest and abdominal CT (17). In 2009, a preoperative prognostic model introduced by Karakiewic et al. for RCC patients treated with nephrectomy exceeded the accuracy of the existing pretreatment models (18). As for metastatic RCC (mRCC), the most used MSKCC model was based on a clinical trial involving patients treated with IFN-α. This model was further developed and now consists of Karnofsky performance status (KPS), serum lactate dehydrogenase (LDH) levels, corrected serum calcium levels and serum hemoglobin levels, and no history of nephrectomy (19). In this era of targeted therapy, Heng proposed another famous prognosis model for mRCC patients, the IMDC model, which played an important role in selecting eligible patients for many clinical trials (20). Although these models have covered all stages of RCC and have a good ability to predict prognosis, some drawbacks still exist. There are few models that specifically focus on stage III RCC patients, and prognosis is rather heterogeneous for these. However, these commonly-used models only incorporate clinical and pathological factors, without considering genetic characteristics. Thus, a more precise prognosis model for stage III RCC patients is necessary.

Recently, some powerful multi-gene signatures in predicting the prognosis of RCC patients were proposed. ClearCode34 is a classifier that can divide localized ccRCC patients into good risk (ccA) group and poor risk (ccB) groups using the expression levels of 34 genes to further analyze patient outcome (21). Additionally, Rini et al. proposed a 16-gene assay to predict recurrence after surgery in localized RCC patients (22). Morgan et al. developed a multi-gene signature based on cell cycle proliferation to improve prediction of mortality within 5 years for RCC patients who underwent radical nephrectomy (23). All three multi-gene signatures showed excellent performance in the training and validation cohorts. However, they all included stage I to stage III renal tumor patients and did not specify locally-advanced RCC patients. In addition, Morgan's signature prediction also included patients with papillary RCC or chromophobe carcinoma. Our study focused on predicting the prognosis of stage III renal cancer patients, and to better apply it to clinical treatment we only included patients with ccRCC. In this respect, our model is more focused and precise in predicting the prognosis of locally advanced clear cell RCC cases according to experimental design. However, whether our signature has predictive advantages over the abovementioned general models remains to be tested in large external stage III clear cell RCC cohorts.

Our model for patients with stage III ccRCC contains nine genes. Among them, *PREX1* was upregulated in RCC tissues in this study. The P-Rex family are Dbl-type guanine-nucleotide exchange factors for Rac family small G proteins and *PREX1* is involved in the inflammatory response. Upregulation of *PREX1* expression occurs in many types of cancers, particularly in breast and prostate cancers and in melanoma (24). In addition, downregulation of *PCSK1N, SELENBP1, SERPINA5*, and *ANK3* was discovered in RCC samples. PROSAAS is a protein encoded by *PCSK1N* (25), and is reported to play an important role in regulating body weight and glucose metabolism as a neuropeptide (25, 26). Thus, downregulation of *PCSK1N* may result in obesity, which is a risk factor for RCC (27). SELENBP1 (selenium-binding protein 1) has already been described as a tumor suppressor involved in the regulation of cell proliferation, senescence, migration, and apoptosis (28). Ha et al. reported that decreased *SELENBP1* mRNA expression is associated with poor prognosis in RCC patients (29). Moreover, this was also confirmed in melanoma, colorectal, breast, prostate, pancreatic, hepatocellular, ovarian, nasopharyngeal, and esophageal carcinoma (28, 30–37). SERPINA5 (protein C inhibitor) is a member of the serine protease inhibitor family and is produced in tissues including the liver, kidney, and testis (38). *SERPINA5* was reported to be deregulated in renal, breast, prostate, liver, and ovarian cancers (38–42) and have a protective role against tumor development, invasiveness, and metastasis (43). Hence, SERPINA5 might be a potential therapeutic target in RCC (38). *ANK3* is mainly expressed in tissues such as kidney and gut epithelium (44), and encodes ankyrin-G isoforms that anchor membrane protein complexes to the cytoskeleton (45). It was discovered that *ANK3* is implicated in renal magnesium handling (46) and polycystic kidney disease (47). Deregulation of *ANK3* expression has been observed in multiple human cancers, and, while it contributes to poor prognosis (48), its mechanism remains unknown (49). Several researchers proposed a possible connection between *ANK3* dysregulation and epithelial-to-mesenchymal transition (EMT) (48). In our future work, we plan to focus on several of the abovementioned genes to determine their roles in RCC patients.

According to the 2018 edition of the EAU guidelines for stage III ccRCC patients, various adjuvant therapeutic strategies are recommended. Thus far, no evidence from randomized phase III trials have confirmed if adjuvant therapy will lead to OS benefit (50). However, some ongoing clinical trials may provide evidence for the future adoption of adjuvant therapy such as new tyrosine kinase inhibitors and nivolumab. One study suggested that full-dose sunitinib could improve DFS in a subset analysis (51, 52). Therefore, we could expect the clinical results of several trials involving adjuvant new TKIs or combined with immune checkpoint inhibitors in the following years.

Few studies have reported laterality as an independent prognosis factor in ccRCC patients (Table 2). We speculated that it was due to the small sample size of the discovery cohort and potential selection bias. Moreover, in the entire set, laterality lost its prognostic power.

Some limitations still existed in this study. Firstly, the nine-gene signature for stage III ccRCC patients was generated using data derived from TCGA and GEO databases, in which most patients were Caucasian, African, or Afro-Caribbean. Secondly, this signature was only validated in the FUSCC cohort. Therefore, this model needs to be further validated in multiple centers across different populations.

## Conclusion

Our study built a nine-gene signature for prognosis prediction in stage III ccRCC patients using data from GSE53757 and TCGA. Results from the validation cohort at FUSCC showed that this model had decent discriminative ability for stage III ccRCC patients and could complement the TNM staging system. However, this signature requires further validation at different centers.

## Data Availability

The datasets generated and analyzed during the current study are available in the GEO database (http://www.ncbi.nlm.nih.gov/geo/) with an identifier GSE53757 and TCGA database obtained from UCSC Xena (https://xenabrowser.net/datapages/). Besides, The datasets from Fudan University Shanghai Cancer Center (FUSCC) are also available in supplementary material.

## Ethics Statement

This study was carried out in accordance with the recommendations of the Research Ethics Committee of Shanghai Cancer Center, Fudan University, China according to the provisions of the Declaration of Helsinki (as revised in Fortaleza, Brazil, October 2013). The protocol was approved by the Research Ethics Committee of Shanghai Cancer Center, Fudan University, China. Written informed consent was obtained from all individual participants included in the validation cohort at FUSCC before they underwent surgery, in accordance with the Declaration of Helsinki. For the public GEO and TCGA databases, we did not need the informed consent of the patients. In addition, patients in the discovery and validation cohort were anonymous and other personal information was also erased.

## Author Contributions

JW and SJ analyzed the data and drafted the manuscript. WG and FW helped interpreted the data. HZ and GS prepared all figures. JW and SJ edited all tables. YQ and DY designed the study. All authors read and approved the final manuscript.

### Conflict of Interest Statement

The authors declare that the research was conducted in the absence of any commercial or financial relationships that could be construed as a potential conflict of interest.

## Abbreviations

ccRCC: clear cell renal cell carcinoma
TCGA: The Cancer Genome Atlas
FUSCC: Fudan University Shanghai Cancer Center
TNM: Tumor Node Metastasis
DFS: disease-free survival
OS: overall survival
GEO: Gene Expression Omnibus
DEGs: differentially expressed genes
ROC: receiver-operating characteristic
HR: hazard ratio
CI: confidence interval
mRCC: metastatic renal cell carcinoma
TKI: tyrosine kinase inhibitor
KPS: Karnofsky performance status
LDH: serum lactate dehydrogenase.
