# Supplementary material for: Construction and Validation of a 9-Gene Signature for Predicting Prognosis in Stage III Clear Cell Renal Cell Carcinoma
Source: Front Oncol. 2019 Mar 19;9:152. doi: 10.3389/fonc.2019.00152 (PMC6433707; doi:10.3389/fonc.2019.00152)
Supplement: Supplementary file 1 [file Data_Sheet_1.docx]

Supplementary Figure 1.

(A) Plot of LASSO coefficient profiles;(B) Plot of partial likelihood deviance for the 1370 common DEGs in TCGA discovery cohort


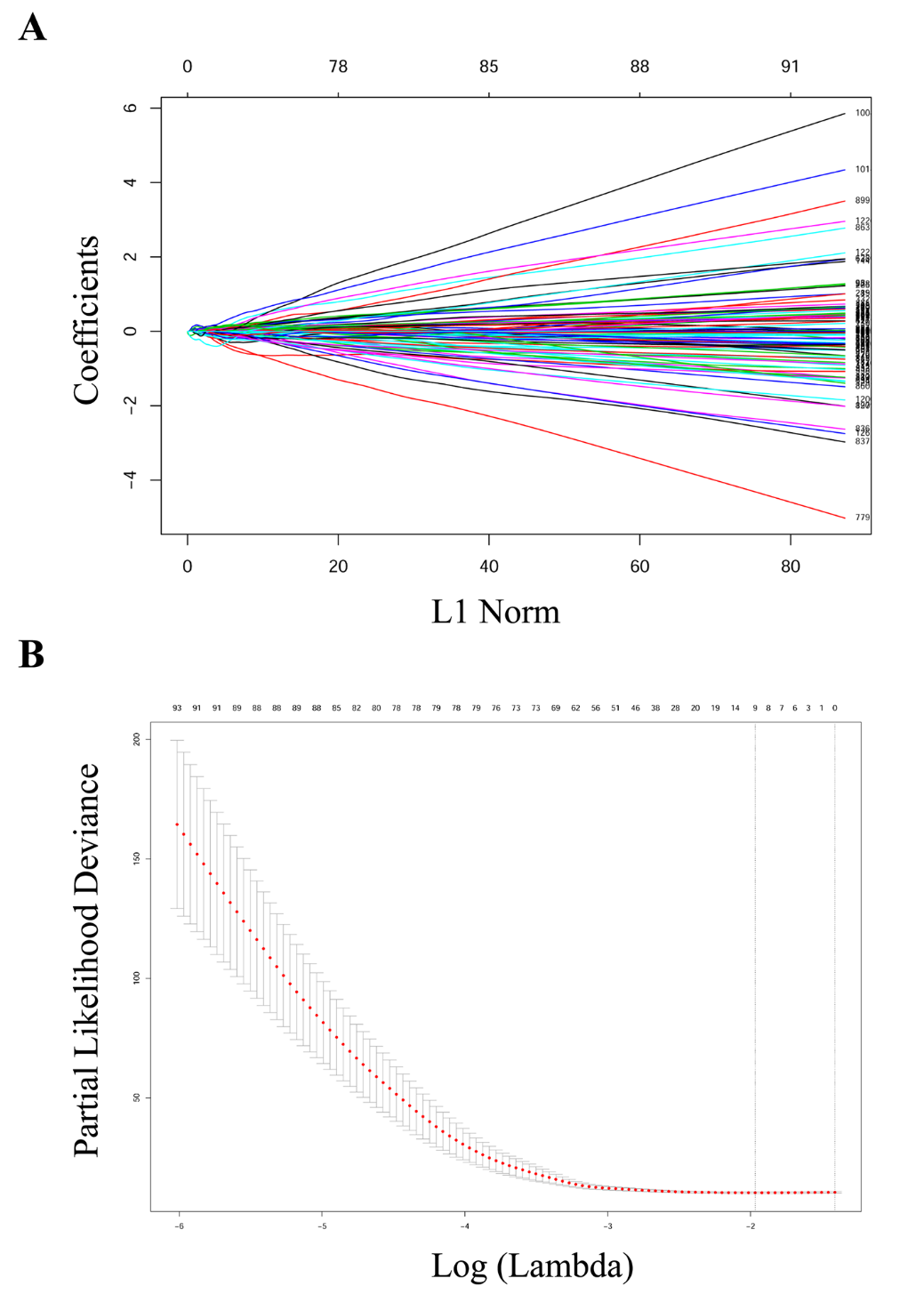


Supplementary Figure 2.

The Kaplan-Meier plot (low risk versus high risk ccRCC cases) of 5 year overall survival in (A)patients older than 60;(B) patients younger than 60;(C)male patients; (D)female patients in the entire set


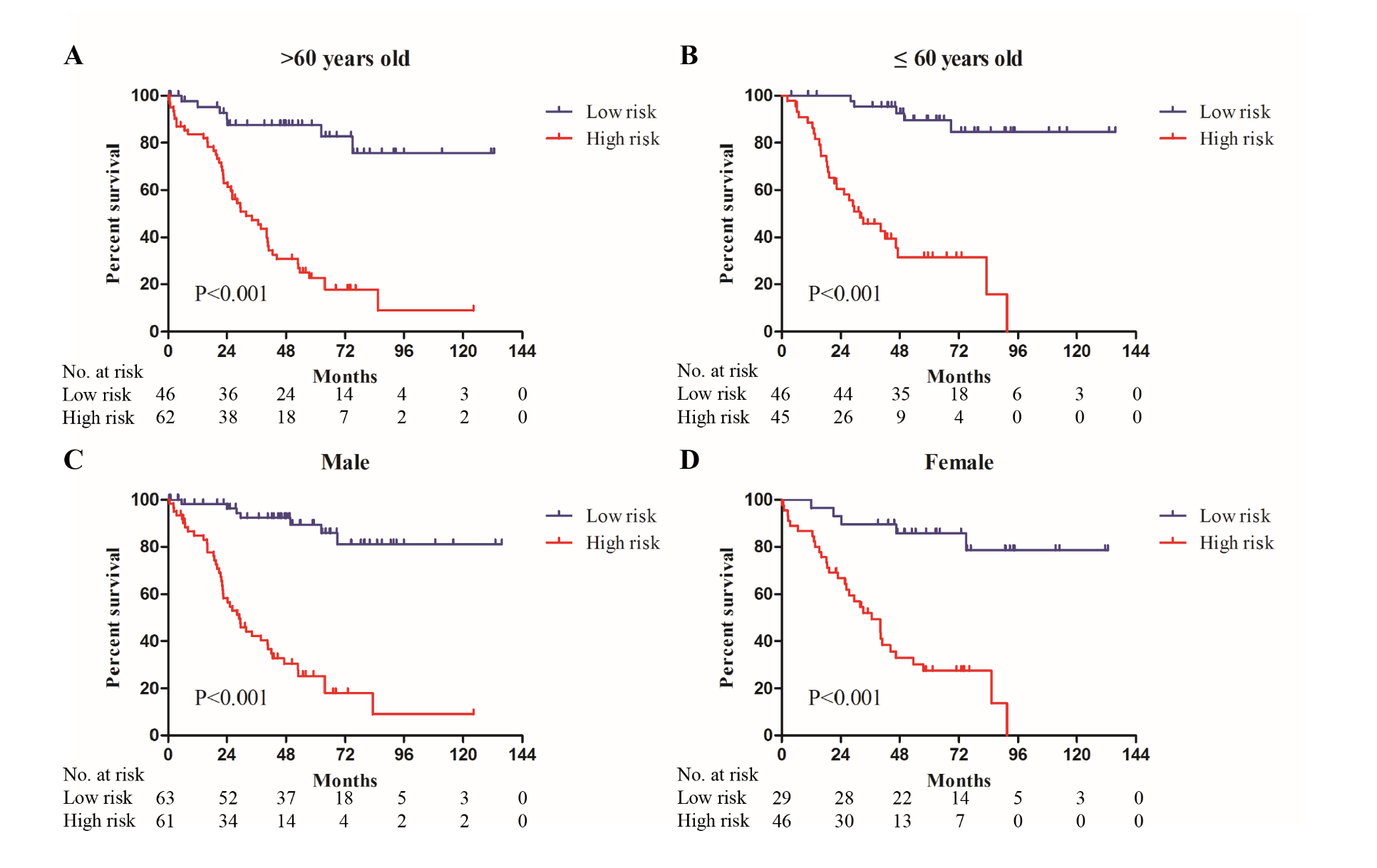


Supplementary Table 1. Primer sequences of nine genes tested in this study.

| Gene | Forward primer, 5’—3’ | Reverse primer, 5’—3’ |
| --- | --- | --- |
| SERPINA5 | AGCAAACGAAGGGCAAGATTG | GGGTGCCTTTGTGGTTGAAG |
| TYRP1 | CCCTGGATATGGCAAAGCG | CCTGTCCTACCCCAAGGAAAG |
| SELENBP1 | TCCCCAGTATTGCCAGGTCAT | CGACTTGGTGCTATCACCGAA |
| ATP6V1C2 | TTTCTGCCCCTGGCGATAAG | AAAGGTGTCGAGTTTCCCCAA |
| PCSK1N | ACCCCGAGCTGTTGAGGTA | GGGTCTCTAGGCGTTTCACA |
| HLA-DRA | TCTGGCGGCTTGAAGAATTTG | GGTGATCGGAGTATAGTTGGAGC |
| GABRA2 | TGCCCAATGCACTTGGAGG | GGAGCAACCTGTACTGAATCAGA |
| ANK3 | GAAGATGCAATGACCGGGGA | CTAAAGCCCATGTAACCCTCTG |
| PREX1 | GGCATTCCTGCATCGCATC | CGGGTGTAAACAATACTCCAAGG |

Supplementary Table 2. Basic characteristics of nine genes tested in this study.

| Gene | Description | Function | Cytoband | UniGene |
| --- | --- | --- | --- | --- |
| SERPINA5 | Serpin Peptidase Inhibitor, Clade A (Alpha-1 Antiproteinase, Antitrypsin), Member 5 | heparin binding and protease binding | 14q32.13 | Hs.159628 |
| TYRP1 | Tyrosinase Related Protein 1 | protein homodimerization activity and oxidoreductase activity | 9p23 | Hs.270279 |
| SELENBP1 | Selenium Binding Protein 1 | selenium binding | 1q21.3 | [Hs.632460](http://www.ncbi.nlm.nih.gov/UniGene/clust.cgi?ORG=Hs&CID=632460) |
| ATP6V1C2 | ATPase, H+ Transporting, Lysosomal 42kDa, V1 Subunit C Isoform 2 | protein dimerization, proton-exporting ATPase activity, phosphorylative mechanism | 2p25.1 | [Hs.580464](http://www.ncbi.nlm.nih.gov/UniGene/clust.cgi?ORG=Hs&CID=580464) |
| PCSK1N | Proprotein Convertase Subtilisin/Kexin Type 1 Inhibitor | signaling receptor binding and endopeptidase inhibitor activity | Xp11.23 | [Hs.522640](http://www.ncbi.nlm.nih.gov/UniGene/clust.cgi?ORG=Hs&CID=522640) |
| HLA-DRA | HLA Class II Histocompatibility Antigen, DR Alpha Chain | peptide antigen binding and MHC class II receptor activity | 6p21.32 | [Hs.520048](http://www.ncbi.nlm.nih.gov/UniGene/clust.cgi?ORG=Hs&CID=520048) |
| GABRA2 | Gamma-Aminobutyric Acid Type A Receptor Alpha2 Subunit | chloride channel activity and GABA-A receptor activity | 4p12 | [Hs.116250](http://www.ncbi.nlm.nih.gov/UniGene/clust.cgi?ORG=Hs&CID=116250) |
| ANK3 | Ankyrin 3, Node Of Ranvier (Ankyrin G) | structural constituent of cytoskeleton and protein binding, bridging | 10q21.2 | [Hs.499725](http://www.ncbi.nlm.nih.gov/UniGene/clust.cgi?ORG=Hs&CID=499725) |
| PREX1 | Phosphatidylinositol-3,4,5-Trisphosphate Dependent Rac Exchange Factor 1 | enzyme bindingand phospholipid binding | 20q13.13 | [Hs.153310](http://www.ncbi.nlm.nih.gov/UniGene/clust.cgi?ORG=Hs&CID=153310) |

Supplementary Table 3. C-Index of clinical factors and 9-gene signature in predicting overall survival in stage III patients.

| Factor |  | Entire set (N=199) | |
| --- | --- | --- | --- |
|  |  | C-Index | 95% CI |
| Age, years |  | 0.590 | 0.524 – 0.655 |
| ISUP grade (I-II vs III-IV) |  | 0.584 | 0.545 – 0.623 |
| pN stage (N0 or Nx vs N1) |  | 0.583 | 0.538 – 0.630 |
| Age + ISUP grade + pN stage |  | 0.690 | 0.634 – 0.746 |
| Nine-gene classifier (Low risk vs High risk) |  | 0.719 | 0.678 – 0.761 |
| All combined (Categorical) |  | 0.792 | 0.749 – 0.835 |

Supplementary File. R-code

> setwd("XXX")

> library(glmnet)

> library(survival)

# Load related packages

> data <- read.csv("File.csv",header = T)

# Load clinical data and RNASeq data

> x <- as.matrix(data[,a:b])

# RNASeq data matrix

> y <- Surv(data$OS,data$Death）

> fit <- glmnet(x,y,family = "cox",alpha = 1,nlambda = 100,thresh = 1e-07)

# Other parameters are default values

> plot(fit,label=T)

> cv.fit <- cv.glmnet(x,y,family = "cox",alpha = 1,lambda = NULL,nfolds = 10)

# Cross-validation for glmnet

> plot(cv.fit)

> a <- coef(cv.fit, s="lambda.min")

# Make predictions and return survived values

> table <- as.matrix(a)

> write.table(table,"table.txt")
